# Assessment of Stimulant Use and Cardiovascular Event Risks Among Older Adults

**DOI:** 10.1001/jamanetworkopen.2021.30795

**Published:** 2021-10-25

**Authors:** Mina Tadrous, Ahmad Shakeri, Cherry Chu, Jennifer Watt, Muhammad M. Mamdani, David N. Juurlink, Tara Gomes

**Affiliations:** 1 Leslie Dan Faculty of Pharmacy, University of Toronto, Toronto, Ontario, Canada; 2 ICES, Toronto, Ontario, Canada; 3 Women's College Research Institute, Women's College Hospital, Toronto, Ontario, Canada; 4 Unity Health Toronto, Toronto, Ontario, Canada; 5 Institute of Health Policy, Management and Evaluation, University of Toronto, Toronto, Ontario, Canada; 6 Sunnybrook Research Institute, Sunnybrook Health Sciences Centre, Toronto, Ontario, Canada

## Abstract

**Importance:**

Use of stimulants continues to increase among older adults for a variety of indications. An association between stimulant use and increased risk of cardiovascular (CV) events has been established among children and young adults, but few studies have explored the risk of CV events among older patients, a group with increased baseline risk.

**Objective:**

To evaluate the association between stimulant use and risk of CV events among older adults.

**Design, Setting, and Participants:**

This propensity score–matched cohort study, with 4 nonusers per 1 user, was conducted from July 1, 2017, to June 27, 2019, using data from population-based health care databases from Ontario, Canada, from January 1, 2002, to December 31, 2016. Included individuals were outpatients aged 66 years or older.

**Exposures:**

Initiation of a prescription stimulant.

**Main Outcomes and Measures:**

The primary outcome was a CV event, defined as a composite of emergency department visit or hospitalization for myocardial infarction, stroke or transient ischemic attack (TIA), or ventricular arrhythmia. Risk of CV event was assessed at 30 days, 180 days, and 365 days after initiation of stimulants from Cox proportional hazard models. A secondary analysis assessed each component of the primary outcome separately.

**Results:**

Among 6457 older adults who initiated a prescription stimulant (ie, the exposed group) and 24 853 older adults who did not initiate such treatment (ie, the unexposed group), the distribution of baseline patient characteristics was well balanced after matching (sex: 3173 [49.1%] men vs 12 112 [48.7%] men; standardized difference, 0.01; median [IQR] age: 74 [69-80] years vs 74 [69-80] years; standardized difference, 0.01). Within this cohort, there were 932 CV events during the 365-day follow-up (5.11 events per 100 person-years among individuals who initiated stimulants). In the primary analysis, stimulant initiation was associated with increased risk of CV events at 30 days (hazard ratio [HR], 1.4; 95% CI, 1.1-1.8) but not at 180 days (HR, 1.2; 95% CI, 0.9-1.6) or 365 days (HR, 1.0; 95% CI, 0.6 to 1.8). In the secondary analysis, stimulant initiation was associated with increased risk of ventricular arrhythmias (HR, 3.0; 95% CI, 1.1-8.7) and stroke or TIA (HR, 1.6; 95% CI, 1.1-2.1) at 30 days.

**Conclusions and Relevance:**

This cohort study found that stimulant use was associated with an early increase in CV events among older adults with no association for long-term use.

## Key Points

**Question:**

Is prescription stimulant use associated with an increased risk of cardiovascular (CV) events among adults aged 66 years and older?

**Findings:**

In this cohort study of 6457 older adults who initiated stimulant use and 24 853 matched older adults who did not initiate such use, stimulant use was associated with a 40% increase in CV events within 30 days of initiation. Risk attenuated over time, with no association with increased risk at 180 days or 365 days after initiation.

**Meaning:**

These findings suggest that safety considerations should be included when stimulants are prescribed to older adults.

## Teaser

This cohort study investigates the association of prescription stimulant use with risk of cardiovascular events among adults aged 66 years and older.

## Introduction

Stimulants act on the central nervous system (CNS) to increase alertness, attention, and energy.^1,2^ Although stimulants are most commonly used among children and youth for the treatment of attention-deficit/hyperactivity disorder (ADHD),^3,4^ an increase in stimulant use among older adults in recent years has been observed.^5^ Off-label uses of stimulants among older adults include the treatment of depression, poststroke recovery, motor function, and fatigue.^2,6,7,8,9,10,11,12,13^ This increase in use has been reported despite concerns regarding cardiovascular (CV) safety.^14,15,16,17,18,19^ Because of their effects on monoamine transmission, stimulants increase resting heart rate and systolic blood pressure. A meta-analysis of 10 randomized clinical trials reported that stimulants were associated with an increase in heart rate of 5.7 beats per minute (bpm) and in systolic blood pressure of 2.0 mm Hg.^14^ Furthermore, the use of methylphenidate was associated with a 4-fold increase in the odds of developing prehypertension among younger adults who were previously normotensive, and use of this stimulant has been associated with increased platelet aggregation.^20,21,22^

To our knowledge, however, few studies have examined the CV safety of stimulants among older adults, a population at inherently increased risk.^23,24,25,26^ This gap in evidence is highlighted as an important area of concern, especially given the increasing use of these agents in this population.^27^ We explored the association between prescription stimulants and the risk of CV events among older adults.

## Methods

The use of data in this cohort study was authorized under §45 of Ontario’s Personal Health Information Protection Act, which does not require review by a research ethics board. Data use without consent is authorized under section §45 of Ontario’s Personal Health Information Protection Act. This study followed the Strengthening the Reporting of Observational Studies in Epidemiology (STROBE) reporting guideline for reporting cohort studies.

### Study Design

The primary study design was a propensity score–matched, population-based cohort study. This methodology was used to account for observed and unobserved confounding factors that may explain the association between stimulant use and risk of CV events.^28,29,30^ To test the robustness of our results, we also conducted 2 sensitivity analyses using a nested case-control design and a case crossover study.

### Data Sources

We used population-based databases documenting health service use and outcomes housed at ICES in Toronto, Ontario. The Ontario Drug Benefit (ODB) claims database, which records prescription medications dispensed to all Ontarians aged 65 years and older, was used to determine exposure to stimulants. We used data pertaining to hospitalizations and emergency department (ED) visits, which were obtained from the Canadian Institute for Health Information Discharge Abstract Database (DAD) and National Ambulatory Care Reporting System (NACRS), respectively. The Ontario Health Insurance Plan (OHIP) Registered Persons Database was used to obtain patient sociodemographic information and dates of death. Demographic and specialty data for all physicians practicing in Ontario were obtained from the ICES Physicians Database, and the OHIP claims database was used to identify claims for all insured physician services. Baseline patient disease history was also documented using corresponding ICES disease-specific registries: the Ontario Congestive Heart Failure data set, Ontario Diabetes Database, and Ontario Hypertension Database^31,32^ These data sets were linked using unique encoded identifiers and analyzed at ICES.

### Cohort Definition

We studied Ontario residents aged 66 years or older from January 1, 2002, to December 31, 2016. We excluded individuals who had invalid patient identifiers, had missing data on age or sex, resided in long-term care facilities, and were receiving palliative care.

The exposure was defined as initiation of a stimulant medication (ie, amphetamine, methylphenidate, lisdexamfetamine, or dextroamphetamine) during the accrual window (ie, January 1, 2002 to March 31, 2015), as evidenced by no other stimulant prescription in the previous 365 days. We defined ongoing use as use of any stimulant, and if a patient switched to a different stimulant but continued stimulant use, they continued to be followed up subsequently. In addition, we defined the dispensing date as the cohort entry date. We created a comparison group of individuals who were not exposed, defined as individuals not prescribed a stimulant over the study period. For these individuals, we randomly assigned index dates to match the distribution of index dates in the exposed group.

We used a high-dimensional propensity score (HDPS) algorithm to generate propensity scores for all patients in the cohort.^28^ The data dimensions used were those available within health claims databases and included the aforementioned data sources for ED visits (NACRS), hospitalizations (DAD), physician billings (OHIP), and prescription drug claims (ODB). We included 7 data dimensions in the algorithm, as follows: all prescription drug claims less than 365 days before cohort entry (1 dimension; ODB), hospitalization and ED diagnoses and procedures less than 365 days before cohort entry (4 dimensions; Canadian Institute for Health Information-DAD diagnoses and procedures, NACRS diagnoses and procedures), and physician service diagnosis and fee codes less than 365 days before cohort entry (2 dimensions; OHIP diagnoses and OHIP fee codes).^33^ Within each data dimension, the 200 most prevalent codes from the previously listed data sets, including diagnostic, procedural, and medication based, were selected; each code was converted into 1 of 3 binary empirical covariates by rating how often a participant got a code: once, sporadic, or frequent.

We selected covariates based on exposure status–covariate associations and not covariate outcomes. This method of covariate selection enabled us to efficiently generate a single matched cohort for all outcomes. In addition, exposure-only selection is associated with improved functionality of the HDPS algorithm when a small number of patients with exposure have an outcome, as was expected in this study.^29^ The top 500 binary empirical covariates were included in the propensity score estimation, including major variables, such as demographic variables (ie, age, sex, income quintile, year of index date, and rural residence), physician visits in the 1 year prior to the index date (including number of physician visits and psychiatrist visits), and CV-associated comorbidities (ie, Charlson Comorbidity Index [CCI] score, diabetes, hypertension, and any CV events in the previous 5 years). We matched each individual who had exposure with 4 individuals without exposure (ie, those in the control group) based on having an HDPS score within 0.2 SDs and age, sex, and history of CV hospitalization (in the prior 5 years).^28,34,35,36^

### Outcome Definition

The primary outcome was any CV event, defined as an ED visit or hospitalization for myocardial infarction, stroke or transient ischemic attack (TIA), or ventricular arrhythmia.^37^ A secondary analysis assessed each component of the primary outcome separately, as well as all cause mortality. Each individual in the cohort was followed up until the first to occur among the primary outcome, death, stimulant discontinuation (defined as a gap between prescriptions that was ≥2-fold the duration of the previously filled prescription), or the end of follow-up (ie, March 31, 2016). For example, if a patient filled a stimulant prescription for 30 days but no follow-up stimulant dispensing was identified within 60 days, we considered them to have discontinued therapy. Their discontinuation date was defined as the date on which their final dispensed prescription ended (in this case, 30 days after dispensing).

### Covariates

We measured baseline demographic characteristics (ie, age, sex, income quintile, and rurality) and indicators of comorbidity, including CCI score (using 3 years of prior hospitalization data) and number of aggregated diagnosis groups in the previous 2 years. Other comorbidity measures captured any time prior to the index date and any CV-associated hospitalization in the past 5 years are listed in eTable 1 in the Supplement. We collected recent medication use data, including the number of distinct drugs dispensed in the past 6 months and indicators of exposure to CV medications (ie, angiotensin-converting enzyme inhibitors, angiotensin II receptor blockers, statins, nonstatin lipid-lowering agents, β-blockers, calcium channel blockers, diuretics, or antiplatelet medications). We used recent use of medications dispensed in the past 6 months because in the province of Ontario it is possible for patients to receive up to a 6-month supply of medication; this allowed us to look at potential concurrent and ongoing medication use. These data were then used in the HDPS and in covariate control. Additionally, we captured use of health services (ie, ED, family physician, cardiologist, or psychiatrist visits) and compared between the 2 groups.

### Statistical Analysis

We conducted a Cox proportional hazards modeling accounting for the matched nature of the data to investigate the association between stimulant initiation and risk of CV events. The proportional hazard assumption was tested, and it did not hold; therefore, we allowed the outcome associated with the exposure to vary over time by including an interaction of time by exposure in the model and estimating the hazard ratio (HR) at multiple times (ie, at 30, 180, and 365 days). We report 95% CIs for each estimate and used a 2-sided test. A significant finding was defined as a 95% CI that did not overlap with 1. We calculated descriptive statistics for patients’ baseline demographic and clinical characteristics and used standardized differences to test for differences between groups. Standardized differences of greater than 0.1 suggest differences between groups for a given characteristic.^38^ All analyses were conducted using SAS statistical software version 9.3 (SAS Institute) from July 1, 2017, to June 27, 2019.

We conducted 2 sensitivity analyses using different observational study designs. In the nested case-control study, individuals meeting inclusion criteria listed previously who had ever received a stimulant constituted the nest and were classified as having a main outcome if they were hospitalized for a CV event during the study period (maximum follow-up date, March 31, 2016). The index date was defined as the date of the individual’s hospital visit. We randomly assigned potential members of the control group an index date based on the distribution of index dates for individuals with the main outcome. All individuals in the nest were eligible to be in the control group regardless of whether they had a main outcome, as long as their assigned index date as part of the control group preceded their outcome date. Individuals with main outcomes and those potentially in the control group were required to have been dispensed at least 1 stimulant in the prior 365 days. We matched up to 4 individuals in the control group with each individual with a main outcome, without replacement, on age (ie, within 3 years), sex, myocardial infarction, or stroke or TIA in the preceding 5 years and diagnosis of hypertension or diabetes in the previous 10 years. Individuals with the main outcome and without at least 1 matched individual in the control group were excluded. Stimulant exposure was classified as current use (defined as receipt of a stimulant prescription within 30 days of the index date), recent use (defined as the most recent stimulant prescription within 31-180 days preceding the index date), and remote use (defined as the most recent stimulant prescription within 181-365 days prior to the index date). We used conditional logistic regression to account for the matched nature of the data, adjusting for the unbalanced baseline characteristics after matching.

In the case-crossover study, we examined only individuals with the main outcome from the nested case-control study described previously. We defined stimulant exposure during hazard and control periods. We assessed whether each individual had any stimulant prescription dispensed during or with a days’ supply overlapping the hazard period (ie, 30 days prior to the index date) and similarly during the primary control period (ie, 90-120 days prior to the index date) and secondary control period (ie, 60-90 days prior to index date). We used conditional logistic regression models to assess the association between stimulant use and risk of CV events.

## Results

We matched 6457 older adults exposed to stimulants with 24 853 older adults who were unexposed, and most baseline characteristics were well balanced (median [IQR] age: 74 [69-80] years vs 74 [69-80] years; standardized difference, 0.01; sex: 3173 [49.1%] men vs 12 112 [48.7%] men; standardized difference, 0.01) (Table 1). The number of individuals with dementia was statistically significantly increased in the exposed group compared with the unexposed group (1814 individuals [28.1%] vs 6365 individuals [25.6%]; standardized difference, 0.06). Furthermore, more individuals in the unexposed group recorded use of angiotensin-converting enzyme inhibitors (7172 individuals [28.9%] vs 1718 individuals [26.6%]; standardized difference, 0.05), statins (11 105 individuals [44.7%] vs 2608 individuals [40.4%]; standardized difference, 0.09), and beta-blockers (6396 individuals [25.7%] vs 1479 individuals [22.9%]; standardized difference, 0.07) in the previous 6 months than those who were exposed to stimulants, but these differences were not statistically significant. Increased use of antipsychotics (1446 individuals [22.4%] vs 4027 [16.2%]; standardized difference, 0.16) and antidepressants (3571 individuals [55.3%] vs 12 660 individuals [50.9%]; standardized difference, 0.09) was found in the exposed group vs the unexposed group, and this difference was statistically significant. There were 69 individuals in the exposed group (1.3%) and 10 individuals in the unexposed group (0.05%) who were exposed to stimulants 1 to 3 years before the index date.

**Table 1.** Baseline Patient Characteristics

Characteristic	Patients, No. (%)		Standardized difference
	With exposure (n = 24 853)	Without exposure (n = 6457)	
Demographic variable			
Age at index date, median (IQR)	74 (69-80)	74 (69-80)	0.05
Sex			
Men	12 112 (48.7)	3173 (49.1)	0.01
Women	12 741 (51.3)	3284 (50.9)	
Income quintile			
1	4623 (18.6)	1196 (18.5)	0
2	4691 (18.9)	1244 (19.3)	0.01
3	4647 (18.7)	1226 (19.0)	0.01
4	4824 (19.4)	1253 (19.4)	0
5	5952 (23.9)	1510 (23.4)	0.01
Missing	116 (0.5)	28 (0.4)	0
Location of residence			
Rural	2680 (10.8)	704 (10.9)	0
Urban	22 148 (89.1)	5746 (89.0)	0
Missing	25 (0.1)	7 (0.1)	0
Measures of comorbidity			
CCI score^a^			
0	3762 (15.1)	968 (15.0)	0
1	2319 (9.3)	579 (9.0)	0.01
≥2	5552 (22.3)	1436 (22.2)	0
No hospitalizations	13 220 (53.2)	3474 (53.8)	0.01
No. of ADGs^b^			
0	4456 (17.9)	1147 (17.8)	0
1-2	12 957 (52.1)	3322 (51.4)	0.01
≥3	7440 (29.9)	1988 (30.8)	0.02
Diagnosis			
Diabetes^c^	7519 (30.3)	1968 (30.5)	0
Hypertension^c^	17 257 (69.4)	4522 (70.0)	0.01
CHF^c^	3577 (14.4)	860 (13.3)	0.03
Fracture^d^	4052 (16.3)	1085 (16.8)	0.01
Dementia^d^	6365 (25.6)	1814 (28.1)	0.06
Any cardiovascular event^d^	2381 (9.6)	625 (9.7)	0
Acute MI event^d^	1014 (4.1)	226 (3.5)	0.03
Stroke or TIA event^d^	1406 (5.7)	410 (6.3)	0.03
Atrial fibrillation event^d^	2070 (8.3)	497 (7.7)	0.02
PVD event^d^	462 (1.9)	116 (1.8)	0
Unstable angina event^d^	843 (3.4)	185 (2.9)	0.03
Health care use			
ED visits, median (IQR)^e^	0 (0-2)	0 (0-2)	0.03
GP or FP visits, median (IQR)^e^	7 (3-12)	6 (3-11)	0.07
Cardiologist visits, median (IQR)^e^	0 (0-0)	0 (0-0)	0.05
Psychiatrist visits, median (IQR)^e^	0 (0-1)	0 (0-1)	0.07
Prescription medication use			
No. of distinct drugs used, median (IQR)^f^	8 (5-12)	8 (5-12)	0.02
ACE inhibitor^f^	7172 (28.9)	1718 (26.6)	0.05
ARB^f^	3630 (14.6)	945 (14.6)	0
Statin^f^	11 105 (44.7)	2608 (40.4)	0.09
Nonstatin lipid-lowering agent^f^	1251 (5.0)	314 (4.9)	0.01
β-Blocker^f^	6396 (25.7)	1479 (22.9)	0.07
Calcium channel blocker^f^	33 (0.1)	7 (0.1)	0.01
Diuretic^f^	7876 (31.7)	1961 (30.4)	0.03
Antiplatelet drug^f^	2666 (10.7)	627 (9.7)	0.03
Cognitive enhancer^f^	2526 (10.2)	626 (9.7)	0.02
Antipsychotic^f^	4027 (16.2)	1446 (22.4)	0.16
Antidepressant^f^	12 660 (50.9)	3571 (55.3)	0.09

Abbreviations: ACE, angiotensin-converting enzyme; ADG, aggregated diagnosis group; ARB, angiotensin II receptor blocker; CCI, Charlson Comorbidity Index; CHF, congestive heart failure; ED, emergency department; FP, family practitioner; GP, general practitioner; IQR, interquartile range; MI, myocardial infarction; PVD, peripheral vascular disease; TIA, transient ischemic attack.

^a^ In the previous 3 years.

^b^ In the previous 2 years.

^c^ Any time prior to the index date.

^d^ In the previous 5 years.

^e^ In the previous 1 year.

^f^ In the previous 6 months.

In the primary analysis, the risk of any CV event among individuals with stimulant use was statistically significantly higher at 30 days after cohort entry compared with those who did not use stimulants (HR, 1.4; 95% CI, 1.1-1.8) (Table 2). This association did not persist at 180 days (HR, 1.2; 95% CI, 0.9-1.6) or 365 days (HR, 1.0; 95% CI, 0.6-1.8). In the secondary analysis examining each component of the outcome separately, we found an association at 30 days between stimulant initiation and risk of arrhythmias (HR, 3.0; 95% CI, 1.1-8.7) and stroke or TIA (HR, 1.6; 95% CI, 1.1-2.1) (Table 2). However, these associations did not persist at 180 or 365 days after cohort entry, except for the risk of arrhythmias at 180 days (HR, 3.0; 95% CI, 1.4-6.4). Stimulant initiation was associated with risk of all-cause mortality in the first 30 days (HR, 2.4; 95% CI, 2.1-2.8); however, the association did not persist at 180 days (HR, 1.0; 95% CI, 0.8-1.2), and there was a statistically significantly decreased risk at 365 days (HR, 0.3; 95% CI, 0.2-0.5). The counts and rate (per 100 person-years) within 1 year for any CV event were 820 events (3.66 events per 100 person-years) and 112 events (5.11 events per 100 person-years) for the unexposed and exposed groups, respectively, for a total of 932 events (eTable 2 in the Supplement).

**Table 2.** High-Dimensional Propensity Score Analysis

Outcome^a^	HR (95% CI)		
	30 d	180 d	365 d
Primary outcome: any CV event	1.4 (1.1-1.8)	1.2 (0.9-1.6)	1.0 (0.6-1.8)
Secondary outcomes			
Acute myocardial infarction	1.0 (0.6-1.7)	1.0 (0.7-1.6)	1.0 (0.4-2.9)
Stroke ore TIA	1.6 (1.1-2.1)	1.3 (0.9-1.7)	1.0 (0.5-1.9)
Ventricular arrhythmia	3.0 (1.1-8.7)	3.0 (1.4-6.4)	2.9 (0.6-14.3)
All-cause mortality	2.4 (2.1-2.8)	1.0 (0.8-1.2)	0.3 (0.2-0.5)

Abbreviations: CV, cardiovascular; HR, hazard ratio; TIA, transient ischemic attack.

^a^ The association between stimulant use and the risk for cardiovascular events is shown, stratified by cumulative duration. The reference group for all comparisons is the unexposed population.

In the nested case-control study, we identified 665 individuals with the main outcome and 1429 matched individuals in the control group. After multivariable adjustment, there was no statistically significant increase in the odds of having a CV event among individuals with current (odds ratio [OR], 0.9; 95% CI, 0.6-2.0) or recent (OR, 1.1; 95% CI, 0.8-1.5) stimulant use compared with those with remote use (eTable 3 in the Supplement). In the case crossover, we found 368 individuals with the main outcome. Similar to the nested case control analysis, the case-crossover study did not have a statistically significant difference in stimulant use during individuals’ hazard period compared with their control period (OR, 0.8; 95% CI, 0.6-1.2) (eTable 3 in the Supplement).

## Discussion

In this population-based cohort study, initiation of prescription stimulants among older adults was associated with a 40% increase in the risk of CV events in the first 30 days after initiation. The HR for this increased risk was highest for ventricular arrhythmia and stroke or TIA. Importantly, we observed no association in the long term (ie, 180 and 365 days after initiation), suggesting attenuated risk over time. These findings come at a time when the use of stimulants continues to increase among older adults and the need for stronger evidence to support safe prescribing in this population is needed.^39,40,41^

Our results suggest the importance of studying drug safety among older adults separately given that meaningful differences exist between this population and others, such as differing pharmacodynamic outcomes and increased prevalence of comorbidities (eg, risk of underlying CV disease).^42^ The evidence gap with prescription stimulant use is even more concerning given the rapid increase in stimulant uptake for a variety of indications.^5,43^ Specifically, in Ontario, the rate of stimulant use among older adults grew by 35% from 2013 to 2017, aligning with similar trends observed in the United States.^39,40,41^ Previous work in Ontario^41^ found that methylphenidate products were the most common stimulant dispensed, followed by lisdexamfetamine, mixed-salt amphetamine, and dextroamphetamine. Long-acting stimulants were the most common formulation dispensed, accounting for 90% of all prescriptions. The increase in stimulant use among older individuals may be associated with off-label use of stimulants (eg, for bipolar depression disorder, fatigue, and cognitive function), and adults living with ADHD are now living longer or being diagnosed later in their lives.^41^ Importantly, despite the increased risk and growing use among this population, to our knowledge, most published studies have largely been limited to children and young adults,^44,45,46,47,48^ and among the few published studies on adults and older adults, the evidence seems to be mixed.^23,24,25,26^ Of 4 observational studies performed among adults, 2 studies were negative and none of these studies studied solely older adults. For instance, a cohort study by Schelleman et al^24^ found a 1.8-fold increase in the risk of sudden death or ventricular arrhythmia among 43 999 new users of methylphenidate; however, more than 90% of study participants were aged younger than 65 years. One study^26^ found an increased risk of heart failure within the first 90 days of initiation among older adults, without a similar finding among younger adults. However, this study explored length of therapy, was limited owing to depletion of patients, and did not compare patients who received treatment with those who did not receive treatment.

Our results may add greater certainty to a mixed body of evidence among the older adult population. These results hold 2 important clinical findings: the first being an increased risk of CV events after the initiation of stimulant use and the second being no association with increased risk with long-term use. The finding of an increased incidence of CV events earlier in the course of treatment is in line with the results of a previous study that examined stimulant use among adults. Mosholder et al^26^ found an increased risk of 1 new heart failure diagnosis per 10.5 person-years of stimulant use, with the symptoms usually appearing within the first 90 days after initiation of the stimulants. Furthermore, known common side effects associated with stimulants described in the literature include tachycardia, arrhythmias, and blood pressure elevation.^49^ The 300% increases in risk for arrhythmias at 30 days (HR, 3.0; 95% CI, 1.1-8.7) and 180 days (HR, 3.0; 95% CI, 1.4-6.4) identified in this study support these literature findings.^50^ On the other hand, our finding that there was no statistically significant increase in CV risk long term contradicts the anticipated hypothesis based on findings from a meta-analysis of 10 randomized, placebo-controlled trials of CNS stimulants that found a statistically significant increased resting heart rate with these stimulants (increase of 5.7 bpm; 95% CI, 3.6-7.8 bpm) and systolic blood pressure (increase of 2.0 mm Hg; 95% CI, 0.8-3.2 mm Hg).^14^ Moreover, given that the trials consisted of younger adult patients with a median (range) age of 36 (22-40) years, we anticipated a greater increase in risk associated with the prevalence of comorbidities among an older population. Findings from our study suggest that perhaps this significant increase in heart rate is not associated with a clinically significant increase in risk. However, our findings must be interpreted with caution given that prescribers may discontinue use among patients who exhibit early signs of CV response (eg, tachycardia or palpitations) while on the medication; thus, there may be a selection bias in which patients who continue long-term treatment are different, which may be associated with a depletion of those at greatest risk. Furthermore, the long-term outcomes associated with minor increased in heart rate among adult patients, particularly patients with concomitant diseases, such as hypertension or heart disease, is largely unknown. Additionally, our work suggests the need to study drug interactions (ie, comedication), other adverse events, and suicidality after stimulant use among older adults.

Although our study is not the first observational study of this question, it is not surprising that the observational evidence has been mixed. This important clinical question presents methodological challenges. Previous studies were cohort studies and produced conflicting results, likely associated with several sources of potential selection bias and the common use of drug trials not captured by data. First, there is a large potential for selection bias among individuals who receive therapy based on current practice trends related to the prescribing of these medications. Prescribers are well aware of the potential risks and may choose to prescribe this treatment only to individuals at the lowest risk of adverse events. Second, it has become common practice for some clinicians to start patients with a short trial to assess the initial response, given that stimulant use appears associated with the greatest risk of adverse cardiovascular events at initiation of therapy. Thus, past experience modifies current risk, which is associated with a depletion of individuals who are susceptible but who are not prescribed psychostimulants a second time. That is, individuals who are susceptible select themselves out of exposure, which is associated with changes in the population at risk, a form of informative censoring. Third, the potential association of stimulant use with cardiovascular health includes short-term and long-term outcomes. The associated increase in short-term risk of tachycardia may occur within hours, while the long-term outcomes of elevated blood pressure may be associated with cardiovascular events over weeks to months. These factors associated with potential selection bias and timing of adverse events suggest that robust and varying study designs should be used, which can help corroborate findings. Fourth, given that discontinuation occurs rapidly in this drug class, the number of individuals using this medication long term is small. Therefore, the absence of associations after 1 year could also be consistent with a loss of statistical power due to a high degree of censoring from medication discontinuation.

For these reasons, our study used the HDPS methodology and sensitivity analyses with varying designs. We used an HDPS matching procedure to create a comparison group closely matched on measured baseline characteristics. This method also attempts to balance individuals on other factors that may be proxies associated with confounders not easily identified in our databases, which may decrease the potential outcomes associated with unmeasured confounding. Furthermore, the incorporation of a nested case-control and case-crossover studies in the sensitivity analyses suggests the robustness of the results and supports the conclusion that ongoing use of stimulants may not be associated with increased risk of CV events. This robust approach allowed us to contrast results of an association that may be difficult to measure owing to challenging methodological factors.

### Limitations

There are several important limitations to our study worth noting. First, we used administrative data and did not have access to relevant clinical variables, such as smoking history, alcohol use, or body mass index. In an attempt to address this shortcoming, we leveraged validated case definitions for individuals living with various comorbidities, including hypertension, and implemented HDPS methodology, which has a benefit of identifying potential proxy variables associated with unmeasured confounders. Second, we had no information on potential drug trials or samples that may have been given prior to the dispensing of initial prescriptions. We suspect that, given the universal drug coverage and inclusion of these agents on the general formulary for individuals ages older than 65 years in Ontario, this is not common practice. Third, according to guidelines, when prescribing methylphenidate, for example, physicians may conduct a controlled discontinuation attempt at least once a year. Thus, patients in our cohort may have been prescribed methylphenidate 2 or 3 years prior to the index date that was successfully discontinued after 1 year. We believe this is not an issue for our study because few patients (69 individuals in the exposed group [1.3%] and 10 individuals in the unexposed group [0.05%]) in our cohort were exposed to stimulants 1 to 3 years before index.^51,52^ Fourth, we did not examine whether there was a dose-response association, for example, whether patients on a higher dose of stimulants were at an increased risk of CV events. However, some guidelines recommend stimulants to be titrated with low doses initially and a slow increase for adults aged 50 years and older.^16^ Because our study examined older adults who were new users, we believe that most of our patients started at low-doses. Fifth, using claims data, we were not able to capture reasons for discontinuation among this patient population.

## Conclusions

In this large population-based cohort of older adults, stimulants were associated with increased short-term risk of CV events, including serious ventricular arrhythmias and stroke or TIA. However, in the long term, stimulants were not associated with an increased risk of CV events, suggesting attenuated risk over time. Our findings do not suggest that clinicians should decrease vigilance in prescribing these agents, but rather suggest that current prescribing practices in patient selection should be continued.

## Notes

Supplement. **eTable 1.** Covariate Codes **eTable 2.** Number of Events and Rate Per 100 Person-Years Within 1 Year From the High-Dimensional Propensity Scores Analysis **eTable 3.** Sensitivity Analyses Results of Associations Between Stimulant Use and Risks for Cardiovascular Events, Stratified by Cumulative Duration
