# Errors in Table, Abstract, and Results

**DOI:** 10.1001/jamanetworkopen.2021.38512

**Published:** 2021-11-17

In the Original Investigation titled “Assessment of Stimulant Use and Cardiovascular Event Risks Among Older Adults,”^1^ published October 25, 2021, there were errors in 2 column headers in a table, an error in a standardized difference in the abstract and results, and 2 standardized differences erroneously referred to as statistically significant in the results. This article has been corrected.^1^
