# Supplementary material for: Assessment of Stimulant Use and Cardiovascular Event Risks Among Older Adults
Source: JAMA Netw Open. 2021 Oct 25;4(10):e2130795. doi: 10.1001/jamanetworkopen.2021.30795 (PMC8546494; doi:10.1001/jamanetworkopen.2021.30795)
Supplement: Supplement. — eTable 1. Covariate Codes eTable 2. Number of Events and Rate Per 100 Person-Years Within 1 Year From the High-Dimensional Propensity Scores Analysis eTable 3. Sensitivity Analyses Results of Associations Between Stimulant Use and Risks for Cardiovascular Events, Stratified by Cumulative Duration [file jamanetwopen-e2130795-s001.pdf]

Supplemental Online Content

Tadrous M, Shakeri A, Chu C, et al. Assessment of stimulant use and cardiovascular event risks among older adults. *JAMA Netw Open*. 2021;4(10):e2130795.
doi:10.1001/jamanetworkopen.2021.30795

eTable 1. Covariate Codes

eTable 2. Number of Events and Rate Per 100 Person-Years Within 1 Year From the High-Dimensional Propensity Scores Analysis

eTable 3. Sensitivity Analyses Results of Associations Between Stimulant Use and Risks for Cardiovascular Events, Stratified by Cumulative Duration

This supplemental material has been provided by the authors to give readers additional information about their work.

© 2021 Tadrous M et al. *JAMA Network Open.*

eTable 1. Covariate Codes

Variable/Condition	Administrative Database	Codes
**Comorbidity codes**		
Dementia	OHIP or DAD and ODB	**ICD-9 Codes**:<br>2900, 2901, 2903, 2904, 2908, 2909, 2948, 2949, 3310-3312, 2941, 797<br>**ICD-10 Code (categorized)**:<br>Alzherimer's Disease- F00.0, F00.1, F00.2, F00.9, G30.0, G30.1, G30.8, G30.9<br>Vascular Dementia - F01.0, F01.1, F01.2, F01.3, F01.8, F01.9<br>Dementia in other disese and unspecified- F02.0, F02.1, F02.2, F02.3, F02.4, F02.8, F03.0, F05.1, F06.5, F06.6, F06.8, F06.9, F09, G31.0, G31.1, R54<br>**OHIP**<br>290, 331, 797<br>**ODB**<br>Claim for cognitive enhancer (DCLASS= 'COGE')
Falls/Fracture	OHIP or DAD	**ICD 9 and 10 codes**:<br>820.xx, S72.0, S72.1, S72.2, 820.x, 812.x, 813.x, 813.x, 813.xx, S42.2, S42.3, S42.4, S42.x, 805.xx, S22.0x, S32.0x, M48.4, M48.4, T08, 807.0, 808.x, 821.x, 733.1x, S72.3, S22.3, S22.4x, S32, S42.8, S42.8, M84.4x, M84.3x, E880-E888, 780.2, 781.2x, 458.0x, W00-W19, R55.xx, I95.1, R26, R27<br>**OHIP**:<br>812, 813, 805, 821, 808, 807
Diabetes	Ontario Diabetes Dataset (ODD)	**ICD 9 and 10 codes**:<br>ICD-9: 250 (any of dxcode)<br>ICD-10: E10, E11, E13, E14 (any of dx10code)<br>**OHIP**<br>Q040, K029, K030, K045<br>**ODB**<br>ODB Claim for DCLASS: INSULIN or ORAL ANTI-GLYCEMICS
Hypertension	Ontario Hypertension dataset (HYPER)	**ICD 9 and 10 codes**:<br>ICD 9 dxcodes: 401x, 402x, 403x, 404x, 405x,<br>ICD 10 dx10codes: I10, I11, I12, I13, I15<br>**OHIP**<br>dxcodes: 401, 402, 403, 404, or 405
**Codes for Cardiovascular Event**		
Acute Myocardial Infarction	CIHI Discharge Abstract Database	ICD9: 410<br>ICD10: I21, I22
Stroke/TIA	CIHI Discharge Abstract Database	ICD9 430,431,434,435,436,362.3<br>ICD10: I60, I61, I63 (excluding I63.6), I64, H34.1, G45 (excluding G45.4), H34.0
Congestive Heart Failure	Ontario Congestive Heart Failure dataset (CHF)	ICD-9: any dxcode='428' (any of dxcode)<br>ICD-10: any dx10code in I500, I501, I509
Peripheral Vascular Disease	CIHI Discharge Abstract Database	ICD9: 441.3,441.4,440.2,443.9,444.2,5012<br>ICD10:<br>I71.3, I71.4, I70.2, I73.9, I74.3, I74.4, IJE57, IJE50, IJE87
Atrial Fibrillation	CIHI Discharge Abstract Database	ICD9: 427.3<br>ICD10: I48
Unstable Angina	CIHI-DAD, inpatient	ICD9: 411, 413<br>ICD10: I20
Ventricular Arrythmia	CIHI Discharge Abstract Database	ICD9: 4271, 4274, 4276, 4278<br>ICD10: I472, I4900

© 2021 Tadrous M et al. *JAMA Network Open.*

eTable 2. Number of Events and Rate Per 100 Person-Years Within 1 Year From the High-Dimensional Propensity Scores Analysis

Association between stimulant use and risk for cardiovascular event, stratified by cumulative duration.

Outcome	Unexposed		Exposed	
	Number of Events	Rate per 100 Person Years	Number of Events	Rate per 100 Person Years
**Primary Outcome**				
Any Cardiovascular Event	820	3.66	112	5.11
**Secondary Outcome**				
Acute Myocardial Infarction	301	1.33	33	1.49
Stroke/Transient Ischemic Attack	517	2.29	78	3.54
Ventricular Arrhythmia	34	0.15	10	0.45
All-cause mortality	1815	7.97	308	13.85

© 2021 Tadrous M et al. *JAMA Network Open.*

eTable 3. Sensitivity Analyses Results of Associations Between Stimulant Use and Risks for Cardiovascular Events, Stratified by Cumulative Duration

**Case-Control Study**		
Sample Size	**Cases**	**Control**
	665	1,429
Primary Outcome<br>*Reference: Remote Use*	**Current Use**<br>OR (95% CI) [a]	**Recent Use**<br>OR (95% CI) [a]
	0.9<br>(0.6-2.0)	1.1<br>(0.8-1.5)
**Case-Crossover Study**		
Sample Size	**Cases**	
	368	
Primary Outcome<br>*Reference: Control Period Use*	**Hazard Period Use**<br>OR (95% CI) [b]	
	0.8<br>(0.6-1.2)	

[a] Current use = stimulant use within 30 days prior to and including index date;
Recent use = stimulant use within 31 to 180 days prior to index date;
Remote use = stimulant use within 181 to 365 days prior to index date.
[b] Control period use = stimulant use either in the 90 to 120 days prior to index date or with a day's supply overlapping the 90 to 120 days prior to index date;
Hazard period use = stimulant use within 30 days prior to index date.

© 2021 Tadrous M et al. *JAMA Network Open.*
